# Chronic Pain Assessments in Children and Adolescents: A Systematic Literature Review of the Selection, Administration, Interpretation, and Reporting of Unidimensional Pain Intensity Scales

**DOI:** 10.1155/2017/7603758

**Published:** 2017-08-21

**Authors:** Rebecca Rachael Lee, Amir Rashid, Daniela Ghio, Wendy Thomson, Lis Cordingley

**Affiliations:** ^1^NIHR Manchester Biomedical Research Centre, Central Manchester University Hospitals NHS Foundation Trust, Manchester Academic Health Science Centre, University of Manchester, Manchester M13 9PT, UK; ^2^Arthritis Research UK Centre for Epidemiology, Centre for Musculoskeletal Research, Manchester Academic Health Science Centre, University of Manchester, Manchester M13 9PT, UK; ^3^Arthritis Research UK Centre for Genetics and Genomics, Centre for Musculoskeletal Research, Manchester Academic Health Science Centre, University of Manchester, Manchester M13 9PT, UK; ^4^Centre for Musculoskeletal Research, Manchester Academic Health Science Centre, University of Manchester, Manchester M13 9PT, UK

## Abstract

**Background:**

Advances in pain assessment approaches now indicate which measures should be used to capture chronic pain experiences in children and adolescents. However, there is little guidance on how these tools should best be administered and reported, such as which time frames to use or how pain scores are categorised as mild, moderate, or severe.

**Objective:**

To synthesise current evidence on unidimensional, single-item pain intensity scale selection, administration, interpretation, and reporting.

**Methods:**

Databases were searched (inception: 18 January 2016) for studies in which unidimensional pain intensity assessments were used with children and adolescents with chronic pain. Ten quality criteria were developed by modifying existing recommendations to evaluate the quality of administration of pain scales most commonly used with children.

**Results:**

Forty-six studies met the inclusion criteria. The highest score achieved was 7 out of a possible 10 (median: 5; IQR: 4–6). Usage of scales varied markedly in administrator/completer, highest anchors, number of successive assessments, and time referent periods used.

**Conclusions:**

Findings suggest these scales are selected, administered, and interpreted inconsistently, even in studies of the same type. Furthermore, methods of administration are rarely reported or justified making it impossible to compare findings across studies. This article concludes by recommending criteria for the future reporting of paediatric chronic pain assessments in studies.

## 1. Introduction

Chronic pain is frequently defined as pain that persists beyond the normal tissue healing time, lasting for three or more months [1], and is estimated to affect between 4 and 40% of children [2]. Changes in reported pain guide treatment decision-making [3] and accurate measurement of pain is associated with improved outcomes in those with long-term chronic conditions [4, 5]. A number of publications recommend that the primary source of information on pain should be the children themselves in paediatric settings [6–8]. However, the developmental changes which occur during childhood and adolescence make the measurement of paediatric pain particularly challenging [6, 9]. The cognitive and metacognitive skills required for a child to give reliable self-reports of pain (such as the ability to rank-order objects, consider numerous options simultaneously, and retain and manipulate information) change significantly during childhood and adolescence [10].

Although assessment of pain in children is complex, there are many single-item pain measures used with this group [11] and unidimensional pain scales are most often used to routinely assess paediatric chronic pain [12]. Whilst they may only provide assessment of one component of pain, these scales are often combined or included in multidimensional and composite pain measurement scales [9]. Some of the most commonly used unidimensional pain scales include visual analogue scales (VASs), numerical rating scales (NRSs), and faces pain scales (FPSs).

Recent attempts to standardise the assessment of pain in children and adolescents with chronic pain include the PedIMMPACT (Paediatric Initiative on Methods, Measurement, and Pain Assessment in Clinical Trials) groups [13] identification of core outcome domains and measures to be used in clinical treatment trials. Other moves towards the standardisation of pain assessment have been made by PROMIS (Patient-Reported Outcomes Measurement Information System) investigators [14] who developed a suite of patient-reported measures to assess a range of chronic conditions, including pain. Whilst these groups' recommendations have been influential in establishing which measures are best to use with children and adolescents with chronic pain, they do not provide corresponding recommendations or advice for how to use and administer pain measures. The lack of standardisation of the approach to pain assessment suggests that methods of measurement may be very different between researchers ostensibly using the same unidimensional pain intensity scales.

King et al. [2] alerted researchers to the problem that inconsistent measurement approaches and reporting pose when establishing the epidemiology of chronic pain in children and adolescents. Other authors have also highlighted that significant bias is created through the different measurement approaches used in paediatric pain research [15, 16]. Research attention has not been given to the variation in the administration of single-item pain assessments or the extent to which inconsistency in measurement approaches may be a problem.

Differences in administration are not the only cause for concern. There is uncertainty about interpretation of pain assessments, particularly when scores are used to classify chronic pain in children and adolescents [17]. The three classifications most commonly used for categorising facets of pain are mild, moderate, and severe. Systems to aid categorisation of pain scores into these classifications have not been well defined in children and few attempts have been made to standardise classification boundaries. Recently, attempts to do this in acute emergency care have been instigated by Tsze et al. [18], who defined ranges of pain scores associated with mild, moderate, and severe pain categories (as measured by the FPS-R and the colour analogue scale (CAS)) in children presenting at emergency departments. Currently, there is no consensus on the points at which pain intensity changes classifications from mild to moderate or from moderate to severe in paediatric chronic pain [17].

To address these issues, a systematic literature review was undertaken to identify, describe, and evaluate current research practices on the selection, administration, interpretation, and reporting of unidimensional chronic pain assessments in paediatric research. The unidimensional pain assessments most commonly used with children and adolescents were reviewed to explore the extent to which differences in research practice exist. From our findings, we develop recommendations for the future use and reporting of chronic pain assessments with children and adolescents.

## 2. Methods

The systematic review is reported according to preferred reporting items for systematic reviews and meta-analyses [19].

### 2.1. Data Sources

Databases searched included Medline (1946–28 December 2015), CINAHL (1937–18 January 2016), Embase (1974–18 January 2016), and PsycInfo (1966–18 January 2016). The date of the last search attempt was 18 January 2016. In addition to database searching, articles were identified through other sources (reference lists of articles and direct contact with authors when articles were irretrievable from databases). Search terms used for the current study included MeSH headings and keywords associated with the following terms: “chronic pain”, “child”, “adolescen^*∗*^”, “pain scale” (as well as specific names of pain measures such as “visual analogue scale^*∗*^”) and “classification” (see Appendix for full search strategy). After these search terms were entered, articles were restricted to English language only. Reference sections of the included studies and review articles were screened for further eligible papers and retrieved where appropriate. One author (RL) conducted the systematic literature search and preliminary screening of article titles/abstracts and identified full-text papers of potential relevance. Two authors (RL and AR) independently reviewed full-text articles for inclusion/exclusion.

### 2.2. Inclusion/Exclusion Criteria

Articles were included if they were studies reporting on the use of a unidimensional pain assessment in children and adolescents experiencing a chronic pain condition (including diagnoses of more specific chronic illness conditions in which pain is a recurring feature, e.g., cancer, headache, and juvenile arthritis), providing that at least one of the primary or secondary outcomes was to assess pain intensity. Studies were included if the children and adolescents in the study sample were between 5 and 18 years of age, similarly to other recent systematic reviews of pain measures used with children and adolescents [20, 21]. Only published peer-reviewed English language, quantitative studies were considered for inclusion. Reviews, commentaries, published abstracts, and qualitative articles were excluded. Studies were excluded if participants who did not have a chronic pain condition formed part of the sample. The purpose of the current review was to explore how pain is assessed in children with existing chronic pain conditions, not how pain assessment tools might be used to screen and identify chronic pain diagnoses (e.g., in community-based epidemiological studies). Where data on children with chronic pain were reported separately, then studies were included.

The unidimensional pain assessments evaluated in the current review include VASs, NRSs, and FPSs as these are the most commonly used with children [22, 23]. VASs are generally considered appropriate for use with children above 7 [24], NRSs for children aged 8 and over [25], and FPSs for children above 4 [26]. Single-item scales used as part of larger multidimensional pain assessments were included and reviewed separately where details were available. However, studies using composite measures were excluded if information on the administration and interpretation of each of the single-item unidimensional scales used within these was not available separately.

### 2.3. Quality Criteria for Pain Scale Selection, Administration, Interpretation, and Reporting

The quality criteria used to assess the selected papers were created by modifying two sets of published recommendations. The first set addressed the issues of selection, administration, and interpretation of pain scales with children and adolescents [8] and the second set addressed clinical practice, education, and research [6]. These sources were chosen as the basis for the development of quality criteria because they were specific to the use of pain assessment tools with children and were not confined to any particular scale or type of pain. A series of three consensus meetings were held by three of the authors prior to the independent critical appraisal of studies to discuss which of these practical points and recommendations would be appropriate to adapt to review the studies included. Authors also discussed the importance of adding quality criteria about the reporting of pain assessments with this group. The more detailed specifications which would need to be met for each criterion to be satisfied were developed, expanded, and agreed on during these meetings (see Table 1 for criteria used and specifications for satisfying the criteria. Criteria which were excluded with corresponding justification are provided in Supplementary Table 1 available online at https://doi.org/10.1155/2017/7603758).

### 2.4. Data Extraction

All the included studies were critically appraised independently by two reviewers against the ten modified quality criteria. In addition to evaluation against quality criteria, a descriptive summary of each of the studies was produced using a systematic approach to data extraction. The summary provided information on each of the following: age range of children included; administrator of the pain assessment; the person completing the pain assessment; anchor points; numbers of successive pain ratings; time/referent period used in the assessment; and classification of pain level produced by the assessment.

## 3. Results

### 3.1. Description of Studies

**Figure 1:**
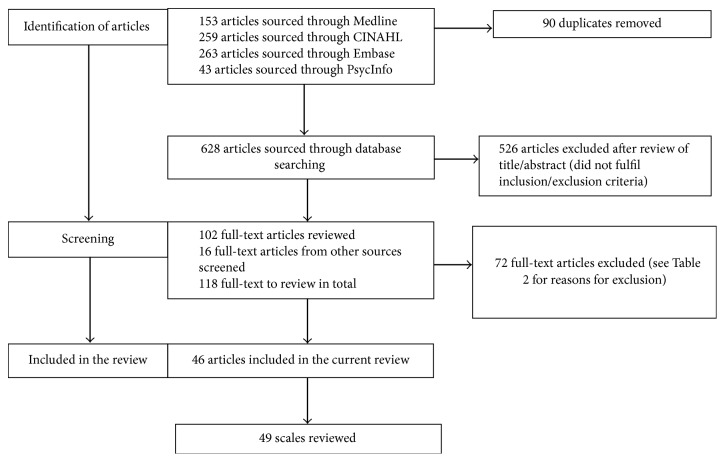
PRISMA flow diagram of study selection process.

The search returned a total of 628 articles for review (after excluding 90 duplicates) and another 16 articles were identified through other sources (see Figure 1). Based on titles and abstracts alone, 526 articles were excluded. For articles of relevance, full-text versions were located and reviewed further. Full-text review of 118 studies resulted in 72 articles being excluded by both reviewers (see Table 2 for justifications). Forty-six papers were included in the final analysis (see Figure 1). Three papers commented on the use of more than one scale [27–29]; hence a total of 49 scales were reviewed. Results reported below are described in relation to either the total number of reported studies (46) or the total number of different scales administered (49).

### 3.2. Excluded Articles

Thirty articles were excluded because they included some study participants who were outside of the specified age range of 5–18. For these studies, it was not possible to identify individual data as it was not reported separately by age. Some of these studies were also excluded because the age range of participants included in the study was not stated, despite the use of the terms “children” or “adolescent” pain assessment in the study title and/or abstract. On closer inspection, the samples included in some of these studies were possibly misclassified as either children or adolescents with some articles including young children aged 3 years and under [30, 31] and young adults up to 22 years old [32].

### 3.3. Quality Criteria

**Figure 2:**
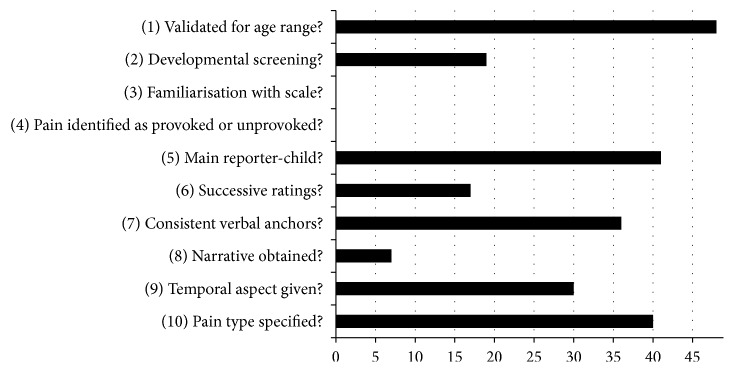
Quality scores against criteria.

Ten quality criteria were used to evaluate the selection, administration, interpretation, and reporting of pain scales meaning each study review resulted in a score of between zero and ten. Two reviewers (RL and AR) read and evaluated the selected studies independently against the quality criteria. On first reading, reviewers achieved consensus on 19 (38.8%). At this point, it was recognised that the main area of disagreement was different interpretations of criterion 2. This was then reassessed and agreement reached on a further 26 studies increasing agreement to over 90%. A third author (LC) independently reviewed the four remaining studies (LC) [15, 17, 33, 34] (see Figure 2).

The maximum score using the quality criteria achieved by the reviewed studies was seven out of a possible ten (median score: 5; IQR: 4–6; see Table 3). The criteria against which most studies scored poorly were conducting developmental screening (19 out of 49 scales), familiarising children with the pain scale (0 out of 49 scales), identification of the nature of the pain measurement taken (provoked or unprovoked) (0 out of 49 scales), conduct of successive ratings of chronic pain (17 out of 49 scales), and gaining a narrative account whilst administering pain assessments (7 out of 49 scales).

### 3.4. Types of Scales

Of the 49 scales, 24 used a VAS, 19 used a NRS, and six used a FPS (3 original, 3 revised versions) (see Tables 4, 5, and 6 for details of administration, interpretation, and reporting information provided in articles).

### 3.5. Administrators of Pain Scales

This section reports the findings from the analysis of 49 scales as reported in 46 studies. Twenty-six of the 40 reports on scales explicitly reported that children and/or adolescents completed the scale themselves [27, 29, 35, 40–43, 48–52, 54, 55, 57–59, 61, 65, 66, 70–72]. Data include that from two in which more than one scale was used [27, 29]. Eleven studies from the included 46 (23.91%) stated that pain measurements were completed with a healthcare professional: seven with psychologists [34, 53, 56, 63, 64, 68, 69], one with a paediatric rheumatologist [54], one with an anaesthesiologist [15], one with a paediatrician or physiotherapist [33], and one not stating which healthcare professional [67].

In ten studies (21.74%) parents completed pain measures in addition to their child's report [29, 35, 40, 42, 48, 54, 55, 58, 59, 72]. These studies reported on the use of nine scales (one study reported the use of more than one scale used with parents and CYP [29]). In two of the included studies (5.71%), only parents reported pain (without an accompanying child report) [39, 44]. In eleven of the included studies (reporting the use of a total of 12 scales) the administrators and completers of assessment were not described at all or reports were ambiguous (23.91%) [17, 28, 36–38, 45–47, 60, 62, 73].

### 3.6. Scale Anchors

Thirty-five studies (76.09%) labelled the “0” anchor as “No Pain” [15, 27, 28, 34, 38–44, 46, 47, 49–54, 56–65, 68, 70]. Three authors described the use of more than one lowest anchor for scales with some using “not hurting,” “no hurt at all,” or “no discomfort” [42, 50, 55]. Other lowest anchors used included “I have no pain” [35] and “none” [45]. Seven studies did not report what “0” signified on the scales used [17, 28, 29, 36, 37, 48, 71]. Eight studies (17.39%) covering nine uses of scales did not report on the highest upper verbal anchor used as an anchor [17, 28, 29, 33, 36, 37, 48, 71]. One of these studies reported two different scale uses [29]. In the remaining articles which did describe highest anchor points, there were 16 variations in wording, namely, “Worst pain” [45, 60, 62, 72], “Worst pain possible” [38, 40, 47, 52, 70], “Very severe pain” [39, 46], “Worst pain imaginable” [27, 28, 41, 43, 44, 49, 53, 57, 66], “Unbearable pain” [27], “A lot of pain” [34], “Most pain possible” [56, 63, 64, 67–69], “Worst pain experienced” [58, 59], “Maximal pain” [61], “Worst pain ever” [65, 73], “I have very severe pain” [35], “Hurting a whole lot” [42, 50, 54, 55], “Severe pain” [42, 50, 54, 55], “Very uncomfortable” [50, 55], “Very much pain” [51], and “The strongest or worst pain you can imagine” [15].

The FPS [75] and the FPS-R [26] have standardised instructions for highest anchor points. For the original FPS, it is recommended that there should not be any written or verbal anchors given to children other than the faces themselves. Two studies in the review discussed the use of top interpretative anchors for children who indicated pain using the highest pain face: “worst pain” [72] and “worst pain ever” [73]. These top anchors were implemented despite no guidance on the interpretation of meaning of pain faces in standardised instructions for this scale. None of the studies which used the FPS-R described the use of written or verbal top anchors although it is suggested by the scale's authors that the top anchor should be verbally described to children as “very much pain.”

### 3.7. Number of Records Kept

Two studies (4.35%) omitted information on the number of successive pain measurements completed [36, 48]. Of the studies that did report this, only 13 studies (28.26%) completed successive ratings of pain [27, 29, 37, 42–44, 46, 50–52, 60–62]. These 13 studies included the use of 15 scales as two studies included the use of two scales [27, 29]. Thirty-two studies (69.57%) explicitly reported taking only one measurement of pain [15, 17, 27, 28, 33–36, 38–41, 45, 47, 49, 53–59, 63–68, 70–73]. These 26 studies reported on the use of 27 scales, with one article reporting only one measurement of pain taken with more than one pain scale [28]. One author described taking successive ratings of pain with a VAS scale but only a one-off assessment with an NRS [27].

### 3.8. Reference Time Frame Captured by Assessments

There were extremely wide variations in the time periods for which participants were due to report on pain. Three studies did not provide details on the reference time given to frame the assessment period [10, 39, 60]. In the remaining 43 studies that did, over 32 different variations were used. Some gave a specific time frame such as “Current pain” [36, 56, 67], “Present pain” [35], “Pain over the past month” [41], “Pain at that moment” [43, 44], “Today” [57], “Daily pain intensity” [50], “How much pain you have right now” [52], “Intensity of pain over the past two weeks” [58], “Pain over the prior two weeks” [62], and “Over the previous four weeks” [72, 73].

Other time frames depended on recollection of specific pain (i.e., worst pain) without a specified time frame: “Average pain” [46, 68], “Usual pain” [71] “Average or usual pain” [65], “Highest, lowest, average pain” [28], “Average, worst, lowest pain” [66], “Most, usual and least pain” [27, 48], “Current pain level and highest pain in the days preceding” [47], “Current, least, average and worst pain intensity” [51], and “Current, lowest and highest pain ratings” [15, 63].

Only 35% (17) of the scales reviewed specified both time frames and specific pain recall: “Typical pain over the past week” [38], “Current pain at rest” [64], “Average daily pain” [69], “Average pain intensity over the previous week” [40, 70], “Most severe and persistent pain” [34], “Mean intensity in the past seven days” [29, 61], “Average of the last seven days” [29], “Best, worst and usual pain” [59], “Maximal pain during the past four weeks” [17], “Present and worst pain intensity for the previous week” [42], “Current, worst and least pain in the last 12 hours” [45], “Present pain and worst pain intensity for the previous week” [54, 55], and “Maximum intensity over the last month” [28, 33].

### 3.9. Classification Details Given in Studies

Only two studies (4.35%) provided information about the classification systems used to categorise pain into mild, moderate, and severe pain categories [17, 61]. This means that 95.65% of articles (44 studies) failed to describe pain classification methods. The two studies which provided information were NRS studies from the same research team [17, 61].

## 4. Discussion

Despite recent attempts to standardise assessment of chronic pain in children and adolescents, little advice exists about how to use and interpret pain measures within paediatric research. Other authors have highlighted how pain is poorly operationalised and approaches to its assessment are inadequately reported across research studies [2, 15, 16, 74] but, to date, there has been no attempt to synthesise evidence from existing pain research studies about how far this problem extends. We conducted a systematic literature review in order to identify, describe, and evaluate paediatric pain assessment research practices with regard to the selection, administration, interpretation, and reporting of chronic pain. The review demonstrates marked variation in the administration of paediatric pain assessments including the administrator and completer of assessments, the anchor points, number of records collected, time/referent periods used to frame assessments, and reported systems for scoring and classification of pain into mild, moderate, and severe pain categories.

Our review identified 46 usable studies which covered 49 reports on the use of unidimensional pain intensity assessments in children and adolescents with chronic pain or conditions in which chronic pain was a feature. Studies were evaluated using a new set of quality criteria devised by the authors which took account of von Baeyer's [6, 8] practical advice on the selection, administration, and interpretation of pain scales and recommendations for clinical practice, education, and research. None of the studies met all ten of the quality criteria. The highest number of criteria met was seven out of ten achieved by only three studies. This highlights the fact that there are currently no guidelines for reporting research use of pain scales with children and adolescents with chronic pain. Importantly, this limits our ability to compare pain outcomes in this patient population. None of the researchers provided evidence that the children were sufficiently familiar with the scale process prior to assessment. Nor did they make it clear whether the children were reporting levels of pain when at rest or when provoked by activity. Studies were marginally better but still poor at recording whether they had conducted developmental screening or collected successive ratings of pain.

In the field of adult pain assessment in analgesic trials, Smith and colleagues found that pain data collection was far from standardised. They concluded that differences in pain assessment methods influenced the inferences drawn in the studies they reviewed [74]. In line with this and other recent commentaries about adult pain assessment [2, 15, 16], the overall picture formed from our analysis is one of tremendous variation in the ways in which researchers assess pain with young individuals with chronic pain. There was no consensus on any aspect of pain assessment administration and interpretation. This review highlights additional issues relating to the administration of pain scales. A significant proportion of studies did not clearly describe either the person administering the assessment or the person completing it. It was difficult to ascertain the degree to which parents were involved in pain reporting in the assessed studies. Around a quarter of all assessments were completed by a parent but still badged as “self-report.” A similar proportion of studies indicated that healthcare professionals were directly involved in the administration of pain assessments to children. Little is currently known about the effect on pain measurement of the presence of a healthcare professional. In most of the studies, pain scales were given to children for independent completion without reference to the degree of cognitive demand involved or to whether it corresponded with the current cognitive capacity of the child [10].

The wide variation in the anchor points used to frame pain assessment is a further source of inconsistency between studies. Even where standardised guidance is available regarding top anchors [75], these recommendations were not always followed [72, 73]. Anchor points can influence pain ratings and currently the potential influence of upper anchors in particular is in need of further study [22]. There was little consensus with some studies using the upper boundary “worst imaginable” pain, whereas others used “worst pain experienced.” A recent editorial advises caution in using worst imaginable pain as an anchor because of the inherent ambiguity for individuals when imagining the “worst possible” pain [76]. Furthermore, the limits of “imagined” pain may be very different depending upon previous pain experiences leading to artificially low pain scores.

Another concern rising from the findings of the current review was the number of studies which based conclusions about children and adolescents with chronic pain upon one-off assessments of the pain episode. Given that this review was based on studies of children with chronic pain (lasting three or more months [1]) and captures pain for children with conditions such as juvenile idiopathic arthritis (which is characterised by intermittent and fluctuating pain episodes [50]), it was a surprise that so many studies limited pain measurement to such a small fragment of the chronic pain experience [8].

A further issue related to timing aspects of pain assessment was that the studies applied widely differing time referent points in their scales. This included different timings on scales of the same type such as VAS or NRS scales. This aspect of pain measure administration highlighted the biggest discrepancy between pain researchers and there was no evidence to support that any one of the referent points was more widely accepted over others. There has been very little exploration of the impact of different time reference points [77] and what the cognitive challenges may be in asking a child or young person to summarize a month's worth of pain experiences into a single response. The complexities involved increase when we take into account the developmental cognitive changes that occur between the ages of five and eighteen years.

In the reviewed studies, pain classification information was rarely presented. Almost all studies failed to report how pain was classified and categorised or what scores were defined as mild, moderate, and severe pain. Many of the studies referred to mild, moderate, and severe pain categories without describing the cut points used to define each category. This has significant implications for consistency and comparability between studies. Scores reported by some authors as indicating moderate pain actually constituted mild or severe pain in others. Overall, results indicate confusion about pain scoring systems [78] which may be in part due to the lack of information reported.

By attempting to collate specific methodological information from studies reporting paediatric pain assessments, the degree of poor or incomplete reporting of the use of pain scales became clear. The majority of studies failed to report basic aspects of measurement procedures and interpretation of pain scores. It was therefore difficult to ascertain whether the use of pain assessments with this population was poor, whether it was good but poorly reported, or both. This review evaluated a wide range of study types from observational to clinical trials and therefore some differences in administrative methods would be expected. However, the extent to which differences occurred within studies of the same nature is problematic. Justifications for differences in selection, administration, or interpretation of pain scales in studies of the same type were not provided. Most importantly, the current situation makes comparisons across pain studies in paediatric research virtually impossible [2, 74].

## 5. Future Directions

Transparent reporting of the use of pain assessments should lead to improvements in the interpretation, reliability, replicability, and comparability of research findings [74]. As a starting point for improving pain assessment administration and reporting in children and adolescents with chronic pain, we suggest that the quality criteria developed for this systematic review are used as guidelines for the reporting of pain assessment tools. These guidelines cover three broad areas: (1) measurement selection, (2) measurement administration, and (3) measurement interpretation. In addition to the modified quality criteria, presentation and interpretation of pain classification information should be provided.

At this stage, there is limited research evidence to suggest that any particular administrative or interpretative methods are better than others. However, we argue that, at the very least by using these criteria and reporting guidelines, researchers will be able to examine and report the differential impacts of methods of pain scale selection, administration, and interpretation. This will enable pain researchers to identify and justify optimal approaches to pain assessment to address their specified research aims. The standardisation of pain assessment methods has been identified as a research priority for reducing bias in pain reports [16, 17]. Standardisation of assessment refers to how measures are used as well as which ones are selected. Our guidelines for the reporting of pain assessments with children and adolescents with chronic pain will go some way towards achieving this aim.

## 6. Conclusions

This systematic review found that the selection, administration, interpretation, and reporting of chronic pain assessments with children and adolescents are inconsistent and poor, and the approaches used are rarely justified. The results of this review provide evidence to suggest that, in paediatric pain assessment, researchers gather information through distinctively different approaches meaning that it is hard to compare and interpret data from different studies. This also demonstrates that there is a weak evidence base on which to base administrative and interpretative decisions about new developing tools. The implications of the findings from this review include the adoption of guidelines for reporting the use of pain assessments with children and adolescents with chronic pain.

## Supplementary Material

Supplementary Table 1: Criteria which were excluded with corresponding justification.

## Figures and Tables

**Table 1:** Quality criteria and specifications.

	Requirements to satisfy this criterion: satisfied if
*Quality of measure selection*	
(i) Is the tool age appropriate?	(i) VAS used for children 7 and above (ii) NRS used for children 8 and above (iii) FPS used for children 4 and above

(ii) Were children screened for developmental delay or was a measure of competency conducted prior to pain assessment?	(i) Studies commented upon completing an assessment for delay prior to conducting the study (ii) They excluded children with developmental delay (assumed they had assessed for this in order to do so)

*Quality of measure administration*	
(i) Did the child have a chance to become familiar with the pain scale used, for example, by rating hypothetical scenarios?	(i) Authors commented upon conducting an assessment of pain prior to the assessment used in the final analysis (ii) Authors explicitly commented upon children and adolescents having time to practice or become familiar with the assessment

(ii) Did authors identify whether pain measurement captured provoked pain levels or pain levels at rest?	(i) Authors specify the nature of the pain measurement captured; for example, do authors comment upon whether pain assessment encompasses pain levels in provoked situations (activity) or unprovoked ones (at rest)?

(iii) Was the child the main reporter of pain at assessment?	(i) Authors explicitly stated that children completed the scale or questionnaire independently (ii) Pain information was gathered as part of a clinical interview with the child or adolescent

(iv) Were successive pain ratings observed?	(i) Authors described taking more than one assessment of pain

(v) Were consistent verbal anchors used across patients in the same study?	(i) Authors described the anchors used

*Quality of measure interpretation*	
(i) Was a narrative explanation of pain scores also obtained, at least at the first data collection point?	(i) Authors explicitly stated that a narrative was gained as part of the pain assessment (ii) Pain assessment was conducted as part of a clinical interview

*Quality of measure reporting*	
(i) Was the temporal frame for pain ratings reported?	(i) Authors state the time scale used to frame pain assessments

(ii) Were the authors clear about which type of pain was measured?	(i) Authors describe the type of pain reported, for example, worst, least, and most pain

**Table 2:** Reasons for exclusion of studies after full-text review of eligibility.

Reason	Number of studies
Not in the defined age bracket (or does not describe age of children/adolescents)	30
Not chronic pain	21
No measure of pain	6
Review or prevalence study	9
Pain assessment tool not described	3
Qualitative study	1
Duplicate	2

**Table 3:** Study characteristics and quality assessments by two independent reviewers.

Tool	Author and year	Chronic pain condition	*N*	Mean age (years)	SD (years)	Female	Range	Quality score /10
VAS	Abu-Saad and Uiterwijk (1995) [35]	Juvenile rheumatoid arthritis	33	—	—	72.3%	7–16	5
VAS	Astfalck et al. (2010) [36]	Chronic low back pain	28	15.55	0.5	50%	14–16	1
VAS	Chan et al. (2009) [37]	Chronic daily headache	12	—	—	—	14–18	2
VAS	Cohen et al. (2010) [38]	Chronic pain	222	14.8	1.9	74.6%	10–18	5
VAS	de Oliveira et al. (2011) [39]	Juvenile idiopathic arthritis	80	11.6	—	42%	8–14	2
VAS	Eccleston et al. (2004) [40]	Chronic pain	80	14.45	1.55	71.25%	11–17	5
VAS	Gold et al. (2009) [41]	Chronic pain	80	13.89	2.57	72.5%	8–18	5
VAS	Gragg et al. (1996) [42]	Rheumatic disease	100	12.2	2.49	68%	8–16	5
VAS	Guite et al. (2011) [27]	Chronic pain	259	15.10	1.64	77.6%	12–18	5
VAS	Hunfeld et al. (2002) [43]	Chronic pain	42	14.5	1.6	—	12–18	7
VAS	Hunfeld et al. (2002) [44]	Chronic pain	85	8.4	2.25	65.88%	5–11	5
VAS	Jacob et al. (2012) [45]	Sickle cell disease	31	13.4	2.4	51.6%	10–17	6
VAS	Kashikar-Zuck et al. (2011) [28]	Chronic pain	1300	14.2	2.4	76%	8–18	5
VAS	Kashikar-Zuck et al. (2012) [46]	Juvenile fibromyalgia	114	15	1.8	92.1%	11–18	4
VAS	Konijnenberg et al. (2005) [47]	Chronic pain	149	11.8	—	73%	8–18	5
VAS	Logan et al. (2006) [48]	Chronic pain	112	15.3	1.3	79.46%	13–18	3
VAS	Lynch et al. (2006) [49]	Chronic back pain	65	14.9	2.6	80%	8–18	6
VAS	Schanberg et al. (2003) [50]	Polyarticular arthritis	41	12.3	2.9	59%	8–17	5
VAS	Stinson et al. (2008) [51]	Juvenile idiopathic arthritis	112	13	2.45	—	8–17	6
VAS	Stinson et al. (2013) [52]	Cancer	47	13.4	—	—	9–18	6
VAS	Swain et al. (2005) [53]	Juvenile fibromyalgia	22	15.62	1.33	100%	13–17	5
VAS	Varni et al. (1987) [54]	Juvenile rheumatoid arthritis	25	9.5	3.17	76%	5–15	7
VAS	Vervoort et al. (2008) [34]	Chronic pain	71	13.33	2.83	57.38%	8–18	4
VAS	Vetter et al. (2014) [55]	Chronic Pain	99	13.2	2.4	71%	8–17	5
NRS	Claar et al. (2008) [56]	Chronic pain	254	14.69	1.49	76.8%	12–17	6
NRS	Cornelissen et al. (2014) [57]	Juvenile idiopathic arthritis	60	13	—	73%	7–17	5
NRS	Guite et al. (2011) [27]	Chronic pain	259	15.10	1.64	77.6%	12–18	5
NRS	Hainsworth et al. (2007) [58]	Chronic pain	60	14.0	2.7	81.7%	8–18	5
NRS	Hainsworth et al. (2009) [59]	Chronic pain	319	13.4	2.7	71%	8–18	4
NRS	Hechler et al. (2014) [29]	Chronic pain	87	14	3.4	74.66%	9–17	6
NRS	Hicks et al. (2006) [60]	Chronic pain	47	11.7	2.1	63.83	9–16	4
NRS	Hirschfeld et al. (2013) [61]	Chronic pain	167	14.1	1.91	62%	11–18	6
NRS	Hirschfeld et al. (2013) [17]	Chronic pain	2249	12.45	2.93	61%	—	2
NRS	Jastrowski et al. (2013) [62]	Chronic pain	6	15	—	—	12–17	4
NRS	Logan et al. (2008) [63]	Chronic pain	414	14.7	1.6	75.6%	12–17	4
NRS	Logan et al. (2013) [64]	Chronic pain	614	13.88	—	74.73%	7–18	5
NRS	Palermo et al. (2008) [65]	Chronic pain	155	14.31	2.45	58.06%	8–18	5
NRS	Ruskin et al. (2014) [15]	Chronic pain	143	14.1	2.4	72%	8–17	6
NRS	Ruskin et al. (2015) [66]	Chronic pain	16	15.75	1.00	100%	13–17	5
NRS	Simons et al. (2010) [67]	Chronic pain	126	15	1.5	82.5%	12–17	5
NRS	Simons et al. (2015) [68]	Chronic pain	321	13.73	2.47	74.8%	8–18	5
NRS	Smith et al. (2015) [69]	Chronic pain	310	13.80	2.69	75.2%	8–17	5
NRS	Vowles et al. (2010) [70]	Chronic pain	209	14.8	1.9	74.6%	10–18	5
FPS	Claar and Walker (2006) [71]	Chronic abdominal pain	596	11.59	2.45	61%	8–17	5
FPS	Palermo et al. (2004) [72]	Chronic pain	189	12.4	2.5	60%	8–16	4
FPS	Palermo and Kiska (2005) [73]	Chronic pain	86	14.75	—	67%	13–16	4
FPS-revised	Hechler et al. (2014) [29]	Chronic pain	87	14	3.4	74.66%	9–17	6
FPS-revised	Kashikar-Zuck et al. (2011) [28]	Chronic pain	1300	14.2	2.4	76%	8–18	6
FPS-revised	Ramstad et al. (2011) [33]	Cerebral palsy	153	14.27	2.88	47.06%	8–18	7

*Highest score achieved*								*7*

*Mean score*								*4.86*

*Standard deviation*								*1.26*

*Median score*								*5*

*Interquartile range*								*4*–*6*

**Table 4:** Descriptive information of VAS studies.

VAS study authors	Age range (mean)	Children completed assessment	Completed with clinician	Parents completed assessment	Person completing not described	Highest anchor	Classification details provided	Successive ratings gained	Reference time period
Abu-Saad and Uiterwijk (1995) [35]	7–16	✓		✓		I have very severe pain	X	No	Present and worst pain
Astfalck et al. (2010) [36]	14–16 (15.55)				X	X	X	X	Current pain
Chan et al. (2009) [37]	14–18				X	X	X	Yes	X
Cohen et al. (2010) [38]	10–18 (14.8)	✓				Worst pain possible	X	No	Typical pain over the past week
de Oliveira et al. (2011) [39]	8–14 (11.6)			✓		Very severe pain	X	No	X
Eccleston et al. (2004) [40]	11–17 (14.45)	✓		✓		Worst pain possible	X	No	Average pain intensity over the previous week
Gold et al. (2009) [41]	8–18 (13.89)	✓				Worst pain imaginable	X	No	Pain over the past month
Gragg et al. (1996) [42]	8–16	✓		✓		Hurting a whole lot or severe pain	X	Yes	Present and worst pain intensity for the previous week
Guite et al. (2011) [27]	12–18 (15.10)	✓				Unbearable pain	X	Yes	Most, usual, and least pain
Hunfeld et al. (2002) [43]	12–18 (14.5)	✓				Worst pain imaginable	X	Yes	Pain at that moment
Hunfeld et al. (2002) [44]	5–11 (8.4)			✓		Worst pain imaginable	X	Yes	Pain at that moment
Jacob et al. (2012) [45]	10–17 (13.4)				X	Worst pain	X	No	Current, worst, and least pain in the last 12 hours
Kashikar-Zuck et al. (2011) [28]	8–18 (14.2)	✓				Worst imaginable pain	X	No	Highest, lowest, and average pain
Kashikar-Zuck et al. (2012) [46]	11–18 (15)				X	Worst possible pain	X	Yes	Average pain severity
Konijnenberg et al. (2005)	8–18 (11.8)				X	Worst pain possible	X	No	Current pain level and highest pain in days preceding
Logan et al. (2006) [48]	13–18 (15.3)	✓		✓		X	X	X	Most, usual, and least pain
Lynch et al. (2006) [49]	8–18 (14.9)	✓				Worst imaginable pain	X	No	Highest, lowest, and average in the last two weeks
Schanberg et al. (2003) [50]	8–17 (12.3)	✓				Hurting a whole lot or severe pain or very uncomfortable	X	Yes	Daily pain intensity
Stinson et al. (2008) [51]	8–17 (13)	✓				Very much pain	X	Yes	Current, least, average, and worst pain intensity
Stinson et al. (2013) [52]	9–18	✓				Worst possible pain	X	Yes	How much pain you have right now
Swain et al. (2005) [53]	13–17 (15.62)	✓	✓ (psychologist)			Worst imaginable pain	X	No	Highest, lowest, and average in the last two weeks
Varni et al. (1987) [54]	5–15 (9.5)	✓	✓ (paediatric rheumatologist)	✓		Hurting a whole lot or severe pain	X	No	Present pain and worst pain intensity for the previous week
Vervoort et al. (2008) [34]	8–18 (13.33)	✓	✓ (psychologist)			A lot of pain	X	No	Most severe and present pain
Vetter et al. (2014) [55]	8–17	✓		✓		Hurting a whole lot or Severe pain or very uncomfortable	X	No	Pain at the present time and at its worst in the past week

*Note*. “X” signifies details not reported.

**Table 5:** Descriptive information of NRS studies.

NRS study authors	Age range (mean)	Children completed assessment	Completed with clinician	Parents completed assessment	Person completing not described	Highest anchor	Classification details provided	Successive ratings gained	Reference time period
Claar et al. (2008) [56]	12–17 (14.69)	✓	✓ (psychologist)			Most pain possible	X	No	Current pain rating
Cornelissen et al. (2014) [57]	7–17	✓				Worst pain imaginable	X	No	On the day of the study
Guite et al. (2011) [27]	12–18 (15.10)	✓				Worst pain imaginable	X	No	Current, most, usual, and least pain
Hainsworth et al. (2007) [58]	8–18 (14.0)	✓		✓		Worst pain experienced	X	No	Intensity of pain over the past two weeks
Hainsworth et al. (2009) [59]	8–18 (13.4)	✓		✓		Worst pain experienced	X	No	Best, worst, and usual pain
Hechler et al. (2014) [29]	9–17 (14)	✓		✓		X	X	Yes	Average of the last seven days
Hicks et al. (2006) [60]	9–16 (11.7)	✓				Worst pain	X	Yes	X
Hirschfeld et al. (2013) [61]	11–18 (14.1)	✓				Maximal pain	✓	Yes	Mean intensity in the past seven days
Hirschfeld et al. (2013) [17]	Range not given (12.45)				X	X	✓	No	Maximal pain during the past four weeks
Jastrowski et al. (2013) [62]	12–17				X	Worst pain	X	Yes	Pain over the prior two weeks
Logan et al. (2008) [63]	12–17 (14.7)	✓	✓ (psychologist)			Most pain possible	X	No	Current, lowest, and highest pain ratings
Logan et al. (2013) [64]	7–18 (13.88)	✓	✓ (psychologist)			Most pain possible	X	No	Current pain at rest
Palermo et al. (2008) [65]	8–18 (14.31)	✓				Worst pain ever	X	No	Average or usual pain
Ruskin et al. (2014) [15]	8–17 (14.1)	✓	✓ (anaesthesiologist)			The strongest or worst pain you can imagine	X	No	Current, usual, lowest, and strongest
Ruskin et al. (2015) [66]	13–17 (15.75)	✓				Worst pain imaginable	X	No	Average, worst, and lowest pain
Simons et al. (2010) [67]	12–17 (14.8)	✓	✓ (type of clinician unclear)			Most pain possible	X	No	Current pain rating
Simons et al. (2015) [68]	8–18 (13.73)	✓	✓ (psychologist)			Most pain possible	X	No	Average pain rating
Smith et al. (2015) [74]	8–17 (range not given)	✓	✓ (psychologist)			Most pain possible	X	No	Average daily pain rating
Vowles et al. (2010) [70]	10–18 (14.8)	✓				Worst possible pain	X	No	Average/usual pain over the last week

*Note*. “X” signifies details not reported.

**Table 6:** Descriptive information of FPS studies.

FPS study authors	Age range (mean)	Children completed assessment	Completed with clinician	Parents completed assessment	Person completing not described	Highest anchor	Classification details provided	Successive ratings gained	Reference time period
Original Faces Pain Scale (Bieri et al., 1990) [75]									
Claar and Walker (2006) [71]	8–17 (11.59)	✓				X	X	No	Usual level
Palermo et al. (2004) [72]	8–16 (12.4)	✓		✓		Worst pain	X	No	Over the previous four weeks
Palermo and Kiska (2005) [73]	13–16 (14.75)	✓				Worst pain ever	X	No	Pain during the previous four weeks

Faces Pain Scale Revised (Hicks et al., 2001) [26]									
Hechler et al. (2014) [29]	9–17 (14)	✓		✓		X	X	Yes	Average of the last seven days
Kashikar-Zuck et al. (2011) [28]	8–18 (14.2)	✓				X	X	No	Maximum intensity over the last month
Ramstad et al. (2011) [33]	8–18 (14.27)	✓	✓ (paediatrician or physiotherapist)			X	X	No	Maximum intensity over the last month

*Note*. “X” signifies details not reported.

## Appendix

### Full Search Strategy Used in Review

1. Child^*∗*^
2. P^*∗*^ediatric
3. Adolescen^*∗*^
4. Juvenile
5. 1 or 2 or 3 or 4
6. Pain  assess^*∗*^
7. Pain  measure^*∗*^
8. Pain scale
9. Visual analogue scale
10. Faces pain scale
11. Numerical rating scale
12. Verbal rating scale
13. VAS
14. NRS
15. VRS
16. 6 or 7 or 8 or 9 or 10 or 11 or 12 or 13 or 14 or 15
17. Chronic pain
18. Musculoskeletal pain
19. Recurrent pain
20. 17 or 18 or 19
21. Scor^*∗*^
22. Classification
23. Mild
24. Moderate
25. Severe
26. Anchor^*∗*^
27. 21 or 22 or 23 or 24 or 25 or 26
28. 5 AND 16 AND 20 AND 27
29. Limit to article (for Embase or journal article in Medline)
30. Limit to English
