# Supplementary material for: Chronic Pain Assessments in Children and Adolescents: A Systematic Literature Review of the Selection, Administration, Interpretation, and Reporting of Unidimensional Pain Intensity Scales
Source: Pain Res Manag. 2017 Aug 21;2017:7603758. doi: 10.1155/2017/7603758 (PMC5585620; doi:10.1155/2017/7603758)
Supplement: Supplementary file 1 — Supplementary Table 1: Criteria which were excluded with corresponding justification. [file 7603758.f1.docx]

**Supplementary Table 1:** Criteria which were excluded with corresponding justification

|  | **Why were these criteria not retained in amalgamated quality criteria?** |
| --- | --- |
| **Older recommendations** |  |
| 1. Were observational measures used for children with cognitive impairment? | This criterion was not retained because the inclusion criteria were based on self-report measures only so any observational measures were excluded. |
| 7. Was pain information considered in treatment planning? | It was inappropriate to judge studies against this criterion because pain treatment was not necessarily an aspect of all studies. This is more of an ethical issue as opposed to an measurement quality issue. |
| **Newer recommendations** |  |
| 1. Have self-report measures been used as a primary source in a hierarchy of pain assessment measures? | This criterion does not measure the quality of the use of the pain scale, but would rather test our inclusion criteria. Penalising studies for this would have been unfair. It was not achievable because we specified including papers of self-reported pain, not observational measures. |
| 1. Were self-report measures of all relevant pain domains used if the clinical situation permitted? | Given we were examining scientific articles about cohorts or large sample of patients, we were unable to comment on whether the clinical situation permitted measurement of relevant pain domains on an individual basis. |
| 1. Were successive pain ratings taken? | This information was captured by one of the older criterion which was amalgamated in the quality criteria used (6. *Were successive pain ratings observed*?). |
| 1. Was a validated pain scale used? | This information was captured by one of the older criterion which was amalgamated with another in the quality criteria used (1. *Has the tool been validated for the age range with which it was used?*). |
| 7. Were pain intensity scores compared across time within individuals but not between? | This criterion would only be appropriate for longitudinal studies. This was excluded because assessments at multiple time points may not have been in the scope of all studies. Penalising some studies for this would have been inappropriate. |
| 1. Did institutions select a small set of scales for regular use? | We excluded this criterion as it reflects more the specific institutional context rather than the quality of pain measurement carried out. Assessing against this says little about the quality of the tools usage, rather it evaluates the clinical setting. |
| 1. Were other measurements taken such as location, temporal, sensory, emotional, functional etc? | This criterion verges into multidimensional pain assessment which we specified would not be included in the review. Evaluating studies against this would have been inappropriate because they were limited by the current inclusion criteria and specific search for studies which used uni-dimensional measures. |
| 1. Were self-reports taken into account when planning treatment? | This criterion was a similar criterion to number 7 in the older recommendations which was omitted earlier. |
